# Chemical composition of barks from *Quercus faginea* trees and characterization of their lipophilic and polar extracts

**DOI:** 10.1371/journal.pone.0197135

**Published:** 2018-05-15

**Authors:** Joana P. A. Ferreira, Isabel Miranda, Vicelina B. Sousa, Helena Pereira

**Affiliations:** Centro de Estudos Florestais, Instituto Superior de Agronomia, Universidade de Lisboa, Lisboa, Portugal; National Taiwan University, TAIWAN

## Abstract

The bark from *Quercus faginea* mature trees from two sites was chemically characterized for the first time. The barks showed the following composition: ash 14.6%, total extractives 13.2%, suberin 2.9% and lignin 28.2%. The polysaccharides were composed mainly of glucose and xylose (50.3% and 35.1% of all monosaccharides respectively) with 4.8% of uronic acids. The suberin composition was: ω-hydroxyacids 46.3% of total compounds, ɑ,ω-alkanoic diacids 22.3%, alkanoic acids 5.9%, alkanols 6.7% and aromatics 6.9% (ferulic acid 4.0%). Polar extracts (ethanol-water) had a high phenolic content of 630.3 mg of gallic acid equivalents (GAE)/g of extract, condensed tannins 220.7 mg of catechin equivalents (CE)/g extract, and flavonoids 207.7 mg CE/g of extract. The antioxidant activity was very high corresponding to 1567 mg Trolox equivalents/g of extract, and an IC50 of 2.63 μg extract/ml. The lipophilic extracts were constituted mainly by glycerol and its derivatives (12.3% of all compounds), alkanoic acids (27.8%), sterols (11.5%) and triterpenes (17.8%). In view of an integrated valorization, *Quercus faginea* barks are interesting sources of polar compounds including phenols and polyphenols with possible interesting bioactivities, while the sterols and triterpenes contained in the lipophilic extracts are also valuable bioactive compounds or chemical intermediates for specific high-value market niches, such as cosmetics, pharmaceuticals and biomedicine.

## Introduction

The *Quercus faginea* Lam. (Portuguese oak) species is native to the Western Iberian Peninsula, and the North African countries of Morocco, Tunisia and Algeria, where it coexists with other oaks such as *Q*. *ilex* L., *Q*. *suber* L., *Q*. *pyrenaica* Willd., and *Q*. *robur* L. Its distribution has become fragmented in the last centuries [1], and there are concerns on a future area reduction with warming and reduced rainfall trends, since drought is the main limiting factor of sub-Mediterranean oaks [2], and specifically of *Q*. *faginea* [3–4].

Although the wood from this species was valued and intensively exploited for naval construction in the XV-XVI centuries [5], nowadays it is not used in a significant extent, even if its potential and environmental and cultural importance are acknowledged. General descriptions of *Q*. *faginea* wood refer good aesthetic appearance, high density, and considerable mechanical strength [6–8].

Recent research efforts have been made to increase knowledge on the growth and wood characteristics of *Q*. *faginea* with the objective to contribute towards its valorization for high-quality end-uses [9–11].

Little is known about the bark of *Q*. *faginea*. One detailed study of bark anatomy and biometric features was made recently: it showed a persistent rhytidome including 3–5 sequential periderms with thin cork layers with a discontinuous development [12]. Knowledge on the bark complex structure and chemical composition allows a more efficient sampling, fractioning and processing towards specific end-uses.

Bark is an important protection component of trees e.g. against fire, frost, fungal diseases or animal injuries, depending on thickness and structure, and therefore a contributor to sustainability [13, 14]. Barks from different tree species are currently left in the forest or burnt in mills of wood, pulp and paper industries [15,16]. In addition to their fuel value, barks are now viewed also as potential resource for biorefineries given their chemical complexity and diversity and several studies are increasing the otherwise rather scarce information on barks [17–24]. They can be a source of high-value chemicals for a variety of areas, from pharmaceutical and bioactive natural compounds to green polymers and bio-based materials [16,17].

In this study, the bark from *Q*. *faginea* mature trees growing in two locations in Portugal in the species distribution area was chemically analyzed regarding the summative chemical composition and the composition of suberin, lipophilic and polar extractives. The antioxidant properties of the bark extracts were also evaluated. It is our goal to contribute to the valorization of *Q*. *faginea* under a full resource approach, and thereby to the strengthening of the species distribution.

## Materials and methods

The samples from the *Quercus faginea* trees were collected in two locations: one stand in the northeast of Portugal (site 1), near Macedo de Cavaleiros and the other stand in the center of Portugal (site 2), near Vimeiro. For the first site, the authority who issued the permission was Instituto da Conservação da Natureza e das Florestas ICNF, and for site 2 the private owner was asked and gave permission. These study does not involve endangered or protected species.

### Sampling

The bark samples were obtained from *Quercus faginea* trees selected from two stands located in the region of the species natural distribution in Portugal. One stand was located near Macedo de Cavaleiros (northeast of Portugal, 41° 30′ N, 07° 01′ W; 554 m altitude; site 1) and the other near Vimeiro (centre of Portugal, 39° 29′ N, 09° 01′ W; 100 m altitude; site 2). The stands resulted from natural regeneration and were unmanaged, mixed and uneven-aged with an average tree age of 40 years (site 1) and 125 years (site 2) [11].

Three trees from each stand were randomly selected for this study. A cross-sectional disc at breast height (1.3 m above ground) was taken and the bark manually removed. The bark samples were air-dried in a well ventilated indoor room, protected from light. The samples were ground individually in a cutting mill (Retsch SM 2000) using an output sieve of 10 mm x 10 mm, followed by one of 2 mm x 2 mm and fractionated with a vibratory system (Retsch AS 200basic) with standard sieves. After sieving, the 40–60 mesh (0.425 mm—0.250 mm) fractions were collected for chemical analysis.

### Summative chemical analysis

Chemical summative analyses included determination of ash, extractives soluble in dichloromethane, ethanol and water, suberin, Klason and acid soluble lignin and the monomeric composition of polysaccharides. All determinations were made with duplicate samples.

Ash was determined by measuring the residue remaining after incinerating the sample overnight in a muffle furnace at 525°C (TAPPI T 211 om-02).

The extractives were determined with procedures adapted from Tappi 204 cm-97, in a soxhlet system successively with dichloromethane, ethanol and water (all supplied by Sigma-Aldrich, ≥99.8% purity, St. Louis, MO, USA), under reflux, during 6h, 16h and again 16h, respectively. The extractives solubilized by each solvent were determined by mass difference of the solid residue after drying at 105 °C and reported as percent of the original sample.

The suberin content was determined in the extractive-free material by use of methanolysis for depolymerization [25]. A 1.5 g sample of extractive-free material was refluxed with a 3% (m/v) solution of NaOCH_3_ in CH_3_OH (100 ml) during 3 h (both supplied by Sigma-Aldrich, 95% and ≥99.8% purity respectively, St. Louis, MO, USA). The sample was filtrated and washed with methanol, and the filtrated residue was refluxed again with 100 ml CH_3_OH for 15 min and filtrated. The combined filtrates were acidified to pH 6 with 2 M H_2_SO_4_ (Merck KGaA, 98% purity, Darmstadt, Germany) and evaporated to dryness. The residues were suspended in water (50 ml) and the products recovered with dichloromethane in three successive extractions (of 50 ml each). The combined extracts were dried over anhydrous Na_2_SO_4_ (ACS Sigma-Aldrich, ≥99% purity, St. Louis, MO, USA), and the solvent evaporated to dryness. The suberin extracts were quantified and the results expressed in percent of the initial dry mass.

Klason and acid-soluble lignin, and carbohydrates contents were determined on the extracted and desuberinized materials. Sulphuric acid (72%, 3.0 ml) was added to 0.35 g of the sample and the mixture was placed in a water bath at 30°C for 1 h, after which it was diluted to 3% H_2_SO_4_ and hydrolyzed for 1 h at 120°C. The sample was vacuum-filtered through a crucible and washed with boiling purified water. Klason lignin was determined as the mass of the solid residue after drying at 105°C (TAPPI T 222 om-02). The acid-soluble lignin was determined on the combined filtrate by measuring the absorbance at 206 nm using a UV/VIS spectrophotometer (TAPPI Useful Method UM 250). The remaining acid solution was kept for sugar analysis.

The composition of polysaccharides was evaluated after hydrolysis by determining the content in neutral monossacharides (rhamnose, arabinose, xylose, galactose, mannose and glucose), uronic acids (galacturonic and glucuronic acids) in the hydrolysate from the lignin analysis using High Pressure Ion-exchange Chromatography with a pulsed amperometric detector (HPIC-PAD). The compounds were separated in a Dionex ICS-3000 system, with an Aminotrap plus Carbopac PA10 column (250 x 4 mm). The content of acetic acid was also determined in the hydrolysate using a High-Pressure Ion-exclusion Chromatography with a UV/Visible detector (HIPCE-UV). The compounds were separated in a Thermo Finnigan Surveyor installed with a Biorad Aminex 87H column (300 x 7.8 mm).

### Ethanol-water extracts composition

Extracts were prepared using approximately 1 g of the bark samples and a solution of ethanol/water (50/50, v/v), with a 1:10 (m/v) solid-liquid ratio for 60 min at 50°C in an ultrasonic bath (Branson 2200 Scientific Support, Inc., Hayward, CA, USA). The sample was filtrated and the supernatant extract was used to determine the contents in total phenolics, flavonoids and condensed tannins. Each assay was performed at least three times and at least three independent replicates were prepared for each standard and sample.

The antioxidant activity of these extracts was also determined using DPPH and FRAP methodologies.

#### Total phenolics

The total phenolic content was determined using a modified Folin-Ciocalteu method [26]. Gallic acid (GA) was used as standard, the experiment was conducted in triplicate.

An aliquot of each bark extract (100 μl) was mixed with 4 ml of Folin-Ciocalteau (1:10 v/v) reagent (Sigma-Aldrich, ≥99.8% purity, St. Louis, MO, USA) and vortexed. After 3–8 min at room temperature, 4 ml of Na_2_CO_3_ (Sigma-Aldrich, ≥99.9% purity, St. Louis, MO, USA) solution (7.5% m/v) were added, vortexed and incubated in a thermostatized water-bath at 45°C for 1 min. The absorbance of the resulting blue colored mixtures was recorded with a spectrophotometer (UV-160A Recording Spectrophotometer, Shimadzu) at 765 nm against a blank containing only water. The same procedure was followed for preparation of the gallic acid (Sigma-Aldrich, ≥99% purity, St. Louis, MO, USA) calibration curve, using seven previously prepared stock standard solutions in the range of 0.014 to 0.762 g/l; the calibration curve of gallic acid was y = 0.0076x+0.0108 (r^2^ = 1.000). The total phenolic content was expressed as milligrams of gallic acid equivalents (GAE) per g of extract.

#### Total flavonoids

Total flavonoid content was determined using a modified aluminum chloride methodology with catechin (CA) as standard [24]. Aliquots (1 ml) of the extract solutions, or catechin standard solution, were taken to 4 ml water and 0.3 ml NaNO_2_ (Sigma-Aldrich, ≥99% purity, St. Louis, MO, USA) solution (5% m/v) and kept during 5 min in the dark. Then 0.3 ml AlCl_3_ (Sigma-Aldrich, ≥99% purity, St. Louis, MO, USA) solution (10% m/v) was added, and after 6 min 2 ml NaOH (Sigma-Aldrich, ≥98.0% purity, St. Louis, MO, USA) solution (4%, m/v) and 2.4 ml water were added sequentially and vigorously shaken. Absorbance was recorded at 510 nm after 30 min incubation, against water (UV-160A Recording Spectrophotometer, Shimadzu). A standard calibration plot was generated using six sequentially and independently prepared stock standard solutions of catechin (Sigma-Aldrich, ≥99.0% purity, St. Louis, MO, USA) with concentration from 0.10 to 1.0 mg/ml. The concentrations of flavonoid in the test samples were calculated from the calibration plot (y = 0.9268x, r^2^ = 0.950) and expressed as mg catechin (CA) equivalent/g of extract.

#### Condensed tannins

Condensed tannins content was determined by the vannilin-sulphuric acid assay using catechin as standard [27]. An aliquot (1 ml) of the extract sample was dried and dissolved in 10 ml of methanol. An aliquot of 1 ml was added to 2.5 ml of vanillin (Sigma-Aldrich, ≥99.0% purity, St. Louis, MO, USA) solution (1% m/v in methanol) and 2.5 ml of sulphuric acid 25% (m/v in methanol), and the volume of 10 ml completed with methanol. The extract samples and blanks (with 1 ml of methanol) were incubated for exactly 15 min. Subsequently, the absorbance was measured at 500 nm using a UV–Vis spectrophotometer (UV-160A Recording Spectrophotometer, Shimadzu). The same procedure was followed for preparation of the catechin calibration plot from standards with concentrations of 10, 20, 40, 60, 80 and 100 mg/l (y = 0.0738x+0.0054, r^2^ = 0.996). The results were expressed as mg catechin (CA) equivalents/g of extract (mg CA/g).

#### Antioxidant activity

The antioxidant activity of the ethanol-water extracts was determined by two methods to cover the various mechanisms of antioxidant action [28,29]: 2,2-diphenyl-1-picryhydrazyl (DPPH), which measures the free radical scavenging capacity, and ferric reducing/antioxidant power (FRAP), which measures the sample’s ferric reducing power.

The DPPH assay was performed using 2,2-diphenyl-1-picrylhydrazyl hydrate (DPPH, Sigma-Aldrich, ≥99.0% purity, St. Louis, MO, USA), a nitrogen centered free radical having an odd electron that changes from purple to yellow when the odd electron is paired in the presence of a radical scavenger to form the reduced DPPH-H [28,29]. The DPPH results are expressed either as IC_50_ value or as Trolox equivalents on a dry extract base.

Different dilutions of the initial extract and of a stock Trolox (Sigma-Aldrich, ≥97.0% purity, St. Louis, MO, USA) solution (0.2 mg/ml) in methanol were prepared. An aliquot of 100 μL of each methanolic solution were added to 3.9 ml of a DPPH methanolic solution (24 μg/ml). The blank sample consisted of 100 μl of methanol added to 3.9 ml of DPPH solution. After 30 min incubation at room temperature in the dark, the absorbance was measured at 515 nm.

The radical scavenging activity of each sample was calculated by the DPPH inhibition percentage as follows: I % = [(Abs_0_-Abs_1_)/Abs_0_]×100, where Abs_0_ was the absorbance of the blank and Abs_1_ was the absorbance in the presence of the extract at different concentrations. The IC_50_ inhibiting concentration represents the concentration of a sample necessary to sequester 50% of the DPPH radicals and was obtained by plotting the inhibition percentage against the extract concentration. The scavenging effect was also expressed as the Trolox equivalent antioxidant capacity (TEAC) determined from the calibration curve with Trolox solution of different concentrations and the percentage of scavenging effect on the DPPH radical.

The ferric reducing antioxidant power (FRAP) assay depends on the reduction of ferric ion into ferrous ion [30]. The FRAP reagent was obtained by mixing 300 mM sodium acetate buffer (pH 3.6), 10 mM TPTZ (tripyridyl triazine, Sigma-Aldrich, ≥98.0% purity, St. Louis, MO, USA) solution and 20.0 mM FeCl_3_.6H2O solution in a ratio of 10:1:1 (volume). An aliquot (100 μl) of extract or standard was then added to 3 ml of the FRAP reagent and the reaction mixture was incubated at 37°C for 30 min. The absorbance was measured at 593 nm in comparison with a blank. Aqueous solutions of known Trolox concentrations in the range of 0–0.5 Mmol/L were used for the calibration, and the results were expressed as Mmol Trolox equivalents/g dry mass.

### Lipophilic extracts composition

The lipophilic extracts that were solubilized from the bark samples with dichloromethane were recovered as a solid residue after solvent evaporation and dried overnight under vacuum at room temperature. Aliquots (2 mg) of each sample were taken and derivatized in 100 μL of pyridine (Sigma-Aldrich, ≥99.8% purity, St. Louis, MO, USA); the compounds with hydroxyl and carboxyl groups were trimethylsilylated into trimethylsilyl (TMS) ethers and esters, respectively, by adding 100 μl of bis(trimethylsily)-trifluoroacetamide (BSTFA, Sigma-Aldrich, ≥99.0% purity, St. Louis, MO, USA). The reaction mixture was heated at 60°C for 30 min in an oven.

The derivatized extracts were immediately analyzed by injection in a GC–MS Agilent 5973 MSD with the following GC conditions: Zebron 7HG-G015-02 column (30 m, 0.25 mm; ID, 0.1 μm film thickness), flow 1 ml/min, injector 280 °C, oven temperature program, 100 °C (1 min), rate of 10 °C/min up to 150 °C, rate of 4 °C/min up to 300 °C, rate of 5 °C/min up to 370 °C, rate of 8 °C/min up to 380 °C (5 min). The MS source was kept at 220 °C and the electron impact mass spectra (EIMS) taken at 70 eV of energy.

The compounds were identified as TMS derivatives by comparing their mass spectra with a GC–MS spectral library (Wiley, NIST), and by comparing their fragmentation profiles with published data [31,32]. For semi-quantitative analysis the area of peaks in the total ion chromatograms of the GC–MS analysis was integrated and their relative proportions expressed as area proportion of the total chromatogram area. Each aliquot was injected in triplicate and results presented by mean (only standard deviation inferior to 5% was considered).

### Suberin composition

Aliquots of the dichloromethane extracts (5 ml) from the suberin depolymerization reaction were taken, evaporated under N_2_ flow and dried at room temperature (r.t.) under vacuum overnight. The samples were derivatized as described above and immediately analyzed by injection in a GC–MS Agilent 5973 MSD with the following GC conditions: Zebron 7HG-G015-02 column (30 m, 0.25 mm; ID, 0.1 μm film thickness), flow 1 ml/min, injector 280°C, oven temperature program, 100°C (1 min), rate of 8°C/min up to 250°C, rate of 5°C/min up to 300°C (5 min), rate of 5°C/min up to 350°C (5 min), rate of 10°C/min up to 380°C (5 min). The MS source was kept at 220°C and the electron impact mass spectra (EIMS) taken at 70 eV of energy.

The compounds were identified and quantified as described above. Each aliquot was injected in triplicate and results presented by mean (only standard deviation inferior to 5% was considered).

### Statistical analysis

All results were expressed as mean and standard deviation (SD). The significance of differences (*p* ≤ 0.05) among the corresponding mean values was determined using one-way analysis of variance (ANOVA) using the *Sigmaplot*^®^ *statistical software* (version *11*.*0*).

## Results and discussion

### Chemical composition

The summative chemical composition of the *Q*. *faginea* bark samples, from the two sites, is summarized on Table 1. No significant differences were found between the trees of site 1 and the trees of site 2. The mean composition was (in % of the oven dry bark): 14.6% ash, 13.2% extractives, 2.9% suberin, 28.2% lignin and 41.1% polysaccharides. Concerning the extractives, the ethanol and water-soluble compounds showed higher proportion (representing 85.6% of the total extractives and 11.3% of the bark) than the dichloromethane extractives (14.4% of the extractives and 1.9% of the bark).

**Table 1:** Chemical composition (% of total o.d. mass) of bark from *Quercus faginea* from sites 1 and 2 and the mean value.

	Site 1	Site 2	Mean±SD
Ash	12.51±3.92	16.61±4.55	14.56±3.60
Extractives	12.23±2.11	14.17±1.22	13.20±1.38
Dichloromethane	1.65±0.32	2.20±0.41	1.92±0.39
Ethanol	5.52±1.30	4.32±1.45	4.92±0.85
Water	5.06±0.59	7.65±1.20	6.36±1.83
Suberin	2.84±0.64	3.03±1.43	2.94±0.13
Lignin	27.98±1.75	28.36±1.00	28.17±0.27
Acid soluble	2.85±0.87	3.27±0.24	3.06±0.29
Klason	25.13±2.23	25.09±0.84	25.11±0.03
Polysaccharides*	44.44±3.24	37.83±0.97	41.13±1.09

* determined by difference of total chemical components

The humidity in the *Q*. *faginea* bark samples from the two sites was determined after 24 h oven-drying at 60 °C and 2 h at 100 °C, corresponding to 13.4–14.2% of bark.

The extractives content of *Q*. *faginea* bark is very similar to that of the sapwood and heartwood of the trees in these two sites [11]. The results are, however, much higher than those found in the bark of other *Quercus* species: 4.6% in *Q*. *robur* [33], 2.1% in *Q*. *petraea* [34], 5–6% in *Q*. *vulcanica* [35], 5.4% in *Q*. *alba*, 6.6% in chestnut oak and 5.8% in *Q*. *stellata* [36]. In a few cases of other species, the bark was separated in phloem and cork: in *Q*. *cerris* rhytidom, the extractives corresponded to 6.5% and 16.7% in the phloem and cork respectively [37], and in *Q*. *suber* to 6.2% and 10.4% respectively [38], and in *Pseudotsuga menziesii* to 28.4% and 29.2% [22].

The suberin content was low, which is consistent with the cellular characteristics of *Q*. *faginea* bark that contains periderms that produce only thin cork layers; therefore, the bark is mostly constituted by the lignocellulosic phloem tissues [12]. In fact, suberin content is in direct relation with the proportion of cork in the bark i.e. barks with more cork will have more suberin. This is the case for instance of species such as *Q*. *suber* [39, 40], *Q*. *cerris* [18, 37], *Pseudotsuga menziesii* [22] or *Q*. *variabilis* barks [24]. In species with a small proportion of cork, the content in suberin is correspondingly low e.g. in the bark of *Pinus pinea* [41] or *Tectona grandis* [42].

Lignin content is relatively high (28.2%). This is justified by the substantial lignification of bark fibers and sclereids [12]. Lignin content is similar to the observed for sapwood and heartwood (28–29%) of trees from the same species [11]. The comparison with barks of other *Quercus* species shows similar range of values to those reported for *Q*. *robur* (25–35%) [33, 43], *Q*. *petraea* (17–30%) [34, 43], *Q*. *alba* and *Q*. *stellata* (14–26%) [36], and *Q*. *vulcanica* (25%) [35].

The carbohydrate composition based on the monosaccharides found in the acid hydrolysates is summarized in Table 2. The major monosaccharide was glucose (over 50% of the total) and xylose (35.1%); rhamnose and arabinose exist in very small amounts and, together with galactose and mannose accounted for only 9% of total monosaccharides) while uronic acids represented 4.6%; no acetylation of the polysaccharides was detected. Comparing to the carbohydrate composition of *Q*. *faginea* wood, glucose and xylose also represented the major monosaccharides (approximately 90% of the total neutral monosaccharides).

**Table 2:** Carbohydrate composition of bark from *Quercus faginea* from sites 1 and 2 in % of total monosaccharides.

	Site 1	Site 2	Mean±SD
Arabinose	4.79±1.22	4.63±1.20	4.71±0.11
Galactose	2.95±0.39	3.23±0.71	3.09±0.20
Glucose	49.21±2.32	51.32±1.53	50.27±1.49
Xylose	38.27±3.17	32.20±3.73	35.14±4.43
Rhamnose	1.00±0.30	1.17±0.31	1.09±0.12
Mannose	0.58±0.51	1.00±0.13	0.79±0.30
Galacturonic acid	2.85±0.70	6.08±2.90	4.46±2.29
Glucuronic acid	0.35±0.01	0.37±0.04	0.36±0.01
Acetic acid	0.00±0.00	0.00±0.00	0.00±0.00

Similar composition was reported for the *Q*. *cerris* rythidome [37] where glucose and xylose also constituted the major monosaccharides identified (48.4% glucose and 27.9–40.3% xylose). Other barks showed a similar pattern e.g. 47.0% and 33.8% of glucose and xylose respectively in *Betula pendula* [21].

Ash content was particularly high. It is known that bark accumulates inorganic materials and their content is usually much higher than in wood which in this species was about 19 times over [11]. Previously, was already observed that crystals and druses occurred profusely in chambered axial parenchyma in *Q*. *faginea* bark as well as large prismatic crystals in sclereids that might may explain these findings [12]. The ash content of *Q*. *faginea* bark was higher than that of other *Quercus* species, namely in the co-occurring cork oaks (0.7%) [19], the American oak, *Q*. *alba* (0.2–1%) [36] and the so-called European oaks (0.3%) [33] and 9–10% [43]. However, the content was similar to that of *Q*. *vulcanica* that reached 13.5% [35].

### Ethanol-water extracts composition

Table 3 shows the yield and composition of the ethanol-water (1:1) extracts concerning total phenolics, flavonoids and condensed tannins content.

**Table 3:** Composition and antioxidant capacity of ethanol-water extracts of bark from *Quercus faginea*.

	Site 1	Site 2	Mean±SD
Extraction yield (%)	7.51±1.22	5.27±1.60	6.39±1.58
Total phenolics (mg GAE/g extract)	539.98±41.72	720.69±47.46	630.33±127.78
Total flavonoids (mg CE/g extract)	181.67±9.15	227.78±363.19	204.72±32.60
Condensed tannins (mg CE/g extract)	259.03±34.39	182.45±21.39	220.74±54.15
Antioxidant capacity (mg TEAC/g extract)	1252.48±126.71	1881.76±285.10	1576.12±444.97
IC_50_ values (μg extract/ml)*	3.01±0.41	2.25±0.09	2.63±0.54
FRAP (mM TEAC/g extract)	3.85±0.70	5.03±0.26	4.44±0.83

*IC_50_ Trolox in ethanol-water 3.81 μg Trolox/ml;

GAE: gallic acid equivalents; CE: catechin equivalents; TEAC: Trolox equivalents antioxidant

The extraction yield under the conditions used was lower than the total polar extractives determined by soxhlet extraction (6.4% vs. 11.3%). This yield difference between extraction processes was also obtained when analysing sapwood and heartwood of *Q*. *faginea* trees [11]. This indicates that the extraction procedure may be optimized to improve extraction yield e.g. by increasing extraction time or temperature.

Phenolic substances are the major constituents of extractives (Table 3), representing 54% to 72% of the *Q*. *faginea* bark ethanol-water extracts (corresponding to 40.5 and 38.0 mg GAE/g of bark). This is similar to the value found for methanol-water extracts of cork from Algerian *Q*. *suber* (787.0 mg GAE/ g extract) [44] as well as for phenolic contents determined in the heartwood of *Q*. *robur*, *Q*. *petraea* and *Q*. *pyrenaica* [12]. The bark of *Q*. *faginea* contains more phenolics than its sapwood (19.5 mg GAE/g of wood) but lower than heartwood (81.8 mg GAE/g of wood) [11].

The extracts were rich in flavonoids and condensed tannins (204.7 and 220.7 mg CE/g of extract respectively) with some differences between sites: the bark extracts from site 1 were richer in condensed tannins than those from site 2 and the opposite was observed concerning flavonoid content. The natural between-tree variability and the tree age difference between site 1 and 2 may contribute to this difference [45] for wood phenolic composition. Overall the *Q*. *faginea* bark content in flavonoids and tannins was much above the values found for sapwood and heartwood [11].

The phenolic richness of the *Q*. *faginea* bark extracts allow to consider a valorization of this material. Plant polyphenols are important free radical scavenging antioxidants because they are able to capture free radicals and chelate metals that could be responsible for promoting lipid peroxidation, while flavonoids may act against several human diseases and as potent antioxidants depending on the molecular structure, the position of the hydroxyl group and other chemical features [46].

#### Antioxidant activity of ethanol-water extracts

The antioxidant capacity of the ethanol-water extracts from *Q*. *faginea* bark was evaluated by measuring the scavenging capacity against the radical DPPH· and by the ferric reducing antioxidant power (Table 3). The bark extracts exhibited high antioxidant activity (IC_50_ of 3.01 μg extract/ml and 2.25 μg extract/ml for site 1 and 2, respectively), when compared to the antioxidant standard Trolox (3.81 μg Trolox/ml).

The reducing ability of the extracts by the FRAP assay was 3.85 mM Trolox/ g extract (0.29 mM Trolox/ g bark) and 5.03 mM Trolox/ g extract (0.26 mM Trolox/ g extract). As expected the reducing ability was higher in the extracts with higher polyphenolic content given their redox properties and so their ability to act as reducing agents, hydrogen donor, singlet oxygen quenchers or metal chelators.

The comparison of the results with literature data must be done cautiously due to differences in methods and calculations. However, it is clear that the extracts of *Q*. *faginea* bark show a very high antioxidant capacity when compared to other extracts: reported by that the IC_50_ value for *Q*. *suber* cork is 2.79 (water extract), 3.58 (methanol extract) and 5.84 (methanol-water extract) (compared with 2.12 μg/ml for ascorbic acid in methanol and 2.46 μg/ml for ascorbic acid in water [47]. This antioxidant activity is similar than that reported for *Eucalyptus sideroxylon* bark ethanol:H_2_O extract in which the IC_50_ value was 2.25 μg/ml, as compared to Trolox (IC_50_ of 2.90 μg/ml) [48]. The scanvenging activity of the hydroalcoholic extract of *E*. *grandis*, *E*. *urograndis* and *E*. *maidenii* barks was also determined and showed comparatively less antioxidant activity, with an IC_50_ values of, respectively, 6.26 μg/ml, 6.14 μg/ml and 8.24 μg/ml compared with 2.17 μg/ml for ascorbic acid [49].

In general, the chemical characteristics of the ethanol-water extracts of bark from *Q*. *faginea* allow considering its use a source of antioxidants in food or cosmetics industries.

### Lipophilic extracts composition

The results of the GC-MS analysis of the non-polar dichloromethane bark extracts of *Q*. *faginea* are summarized in Table 4.

**Table 4:** Composition of dichloromethane extracts of *Quercus faginea* bark, in % of the normalized chromatographic peak areas in TIC of the compounds detected by GC-MS.

Compounds	Site 1	Site 2	Mean±SD
***Aromatics***	***14*.*8±5*.*7***	***10*.*7±5*.*9***	***12*.*9±5*.*8***
Vanillin	2.3±0.2	1.6±1.4	2.0±0.8
Vanillic acid	5.0±0.4	3.8±1.5	4.4±1.0
Ferulic acid	5.7±4.9	4.4±2.0	5.1±3.4
2,3-Dihydroxy-1-(4-hydroxy-3-methoxyphenyl)-propan-1-one	1.8±0.2	0.9±1.0	1.4±0.6
***Glycerol and derivatives***	***159*.*5±131*.*9***	***78*.*2±56*.*9***	***122*.*9±94*.*4***
Glycerol	113.0±104.9	13.2±12.6	63.1±58.8
1-Monohexadecanoylglycerol	19.1±4.8	20.1±10.0	19.6±7.4
1-Monooctadecanoylglycerol	10.1±1.2	5.9±4.1	8.0±2.6
Monoacylglycerol C24	7.7±4.4	8.1±7.0	7.9±5.7
Monoacyl glycerol C26	9.6±16.6	38.9±23.2	24.3±19.9
***1-Alkanols***	***16*.*7±5*.*9***	***17*.*9±5*.*8***	***17*.*2±7*.*8***
1-Hexadecanol	0.3±0.3	0.5±0.5	0.4±0.4
1-Octadecanol	0.9±0.6	3.9±1.7	2.4±1.2
1-Eicosanol	2.5±2.5	5.1±1.3	3.8±1.9
1-Tetracosanol	8.6±1.9	4.5±1.0	6.6±1.4
1-Tricosanol	4.4±0.6	3.9±1.3	4.2±1.0
***Saturated alkanoic acids***	***304*.*1±62*.*6***	***162*.*7±36*.*0***	***233*.*7±48*.*8***
Nonanoic acid	1.7±0.2	0.5±0.5	1.1±0.4
Decanoic acid	-	0.1±0.1	0.1±0.1
Dodecanoic acid	1.3±1.5	1.2±0.6	1.3±1.1
Tetradecanoic acid	3.6±0.5	3.9±1.7	3.8±1.1
Pentadecanoic acid	2.5±0.2	2.7±0.7	2.6±0.4
Hexadecanoic acid	194.7±45.1	73.7±20.8	134.2±33.0
Heptadecanoic acid	3.3±0.3	3.1±0.8	3.2±0.6
Octadecanoic acid	26.1±0.9	18.7±4.7	22.4±2.8
Eicosanoic acid	8.7±0.9	4.9±0.2	6.8±0.6
Heneicosanoic acid	5.1±1.7	3.2±1.2	4.2±1.4
Docosanoic acid	29.0±3.1	17.3±1.2	23.2±2.2
Tricosanoic acid	4.4±1.0	4.3±1.0	4.4±0.5
Tetracosanoic acid	21.7±3.8	23.0±0.6	22.4±2.2
Hexacosanoic acid	2.0±3.4	6.1±1.9	4.0±2.4
***Substituted alkanoic acids***	***50*.*4±30*.*6***	***38*.*9±27*.*1***	***44*.*7±28*.*8***
9-Hexadecenoic acid	1.2±0.1	0.7±0.6	1.0±0.4
9.12-Octadecadienoic acid	12.4±7.5	9.7±2.4	11.0±5.0
11-Octadecenoic acid	33.9±22.8	26.8±22.4	30.4±22.6
13-Octadecenoic acid	2.9±0.2	1.7±1.7	2.3±0.8
***Saturated ω-hydroxyacids***	***6*.*2±3*.*4***	***8*.*2±5*.*6***	***7*.*2±4*.*6***
22-Hydroxydocosanoic acid	5.2±1.7	2.9±3.0	4.0±2.4
24-Hydroxytetracosanoic acid	1.0±1.7	5.3±2.6	3.2±2.2
***Saturated ɑ*,*ω-alkanoic diacids***	***11*.*2±5*.*0***	***2*.*3±1*.*0***	***6*.*7±2*.*9***
Octanedioic acid	1.2±1.2	0.6±0.1	0.9±0.6
Nonanedioic acid	7.9±2.0	1.7±0.9	4.8±1.4
Hexadecanedioic acid	2.1±1.8	-	1.0±0.9
***Sterols***	***27*.*8±6*.*6***	***202*.*8±120*.*4***	***118*.*1±63*.*5***
Campesterol	-	1.5±2.5	0.8±1.2
Stigmasterol	0.6±0.1	11.9±11.4	9.0±5.8
β-Sitosterol	27.2±6.5	168.4±85.9	97.8±46.2
Stigmastanol	0.00±0.00	21.0±20.6	10.5±10.3
***Triterpenes/Triterpenoids***	***160*.*6±100*.*2***	***195*.*4±129*.*4***	***178*.*0±114*.*7***
β-Amyrin	18.6±6.6	14.9±12.9	16.8±9.8
α-Amyrin	3.3±5.7	21.3±24.4	12.3±15.0
Friedelin	51.1±35.2	64.4±36.4	57.8±35.8
Lupeol	12.0±3.5	26.1±19.4	19.0±11.4
Olean-18-ene	20.7±25.3	23.4±13.6	22.1±19.4
Betulin	15.1±6.4	6.9±5.3	11.0±5.8
Betulinic acid	18.7±10.5	30.0±12.1	24.4±11.3
Ursolic acid	21.1±7.0	8.4±5.3	14.8±6.2
***Identified***	***741*.*3±351*.*9***	***717*.*1±388*.*1***	***741*.*4±371*.*3***
Non Identified	258.7	282.9	258.6
Total	1000.0	1000.0	1000.0

Triterpenes constitute one of the most abundant class of compounds (16.1% and 19.5% of all compounds, respectively for site 1 and site 2). Among triterpenes, friedelin (5.1–6.4% of all compounds), olean-18-ene (2.1–2.3%) and betulinic acid (1.9–3.0%) constitute 90.1% of all the identified triterpenes/triterpenoids. β-Amyrin, ɑ-amyrin, betulin and ursolic acid) were also identified in smaller amounts.

Several authors have reported the presence of betulin, betulinic acid, lupeol and oleanolic acid in birch bark species [50, 51] and of friedelin, cerin, lupeol, ursolic acids in oak barks [49–54]. Barks from *Eucalyptus* species are also very rich in triterpenic compounds as ɑ-amyrin, β-amyrin, betulonic and betulinic acids, oleanolic and ursolic acids [55, 15].

Saturated alkanoic acids constitute also one abundant group of compounds (30.4% and 16.3% of all compounds for site 1 and site 2 respectively). They are the major compounds identified in the lipophilic bark extracts from the trees from site 1, namely hexadecanoic, octadecanoc and docosanoic acids, corresponding to 19.5%, 2.6% and 2.9%, respectively. In site 2, saturated alkanoic acids were also found in considerable amounts, with hexadecanoic, octadecanoic and docosanoic acids also as the most representative (7.4%, 1.9% and 1.7%, respectively). Substituted alkanoic acids, saturated ω-hydroxyacids and ɑ,ω-diacids were also found in the lipophilic extracts from trees from both sites, but in smaller amounts (5.1%, 0.6% and 1.1% for site 1, respectively, and 3.9%, 0.8% and 0.2% for site 2, respectively). Alkanols comprise only 1.7–1.8% of all compounds, with eicosanol and tricosanol as major compounds. Chain lengths vary from C9 to C26, but C16 is the most abundant, corresponding to 53.6% and 45.1% of the total fatty acids, in the lipophilic extracts from sites 1 and 2, respectively.

The same pattern of composition was found for other lipophilic hardwood bark extracts like birch trees [23, 56] or *Populus balsamifera* [57].

Glycerol and glycerol derivatives constituted 16.0% of all compounds in the bark lipophilic extracts from site 1, with glycerol representing 11.3%. In site 2, the amount of glycerol and its derivatives was considerably lower (8.6%).

Sterols were identified in high amounts but only in trees from site 2, constituting 20.3% of all compounds, almost tenfold the amount found in site 1 (2.8%). β-sitosterol was the major sterol (16.8% of all compounds); stigmasterol, stigmastanol and campesterol were also identified in smaller amounts.

Aromatics were present (1.5–1.1% of all compounds) including ferulic acid although in small amounts (<1%).

There are some clear differences in the composition of the bark lipophilic extracts from both sites: in extracts from site 1, the saturated alkanoic acids are clearly the most abundant group (30.9% vs. 16.3%), while for site 2, the major group is the sterols (20.3% vs. 2.8%).

Lipophilic bark extracts from *Q*. *faginea* trees are different from those reported for the wood of the same trees [11]. The composition was not influenced by the geographical location. Aromatics are the major class of compounds in sapwood (22.8%) and also saturated alkanoic acids (15.7%) and sterols (10.6%); in heartwood, saturated alkanoic acids represent the major class of identified compounds (25.8%), accompanied by triterpenes (13.0%), sterols (12.8%) and substituted alkanoic acids (10.4%).

### Suberin composition

The results for the suberin composition obtained by GC-MS analysis are summarized in Table 5, given in mg of compound per kg of dry mass. This is the most commonly used quantification of suberin monomers in the depolymerized mixtures [58]. The mixtures contain the compounds that are soluble in the low polarity organic solvent used for the recovery of the suberin monomers after depolymerization by partition between water and an organic phase after acidification of the reaction mixture; water soluble monomers, namely glycerol are removed in the aqueous phase [25].

**Table 5:** Composition of suberin extracts of *Quercus faginea* bark, in % of the normalized chromatographic peak areas in TIC of the compounds detected by GC-MS.

Compounds	Site 1	Site 2	Mean±SD
***Aromatics***	***97*.*6±29*.*2***	***40*.*1±15*.*7***	***69*.*1±23*.*1***
Vanillin	1.7±0.1	-	0.9±0.1
Vanillic acid	2.8±1.2	-	1.4±0.6
Vanillyl alcohol	0.6±0.3	-	0.3±0.2
Methyl vanillate	2.6±0.8	-	1.3±0.4
Ferulic acid	28.7±21.0	-	14.4±11.0
Ferulic acid, methyl ester	58.2±3.9	40.1±15.7	49.2±9.8
Syringaldehyde	1.0±0.6	-	0.5±0.3
Coniferyl aldehyde	0.3±0.3	-	0.2±0.2
3,5-Dimethoxymandelic acid	1.8±1.0	-	0.9±0.5
***1-Alkanols***	***67*.*5±4*.*8***	***66*.*1±25*.*9***	***67*.*0±26*.*6***
1-Tetradecanol	4.1±0.53	-	2.1±4.4
1-Pentadecanol	7.6±0.16	6.2±2.6	6.9±0.7
1-Octadecanol	5.6±0.36	28.2±8.2	16.9±3.2
1-Eicosanol	9.7±0.05	18.0±7.3	13.9±4.8
1-Docosanol	12.6±2.3	13.7±7.8	13.2±3.9
1-Tetracosanol	2.79±1.4	-	14.0±9.6
***Saturated alkanoic acids***	***65*.*0±24*.*4***	***31*.*5±17*.*5***	***46*.*9±17*.*1***
Tetradecanoic acid	4.1±2.6	-	2.1±1.3
Hexadecanoic acid	6.4±2.8	13.9±6.2	10.2±4.5
Hexadecanoic acid, methyl ester	10.2±5.0	-	5.1±2.5
Octadecanoic acid	4.1±1.2	15.3±7.3	9.7±4.2
Eicosanoic acid	2.7±2.3	-	1.4±1.2
Docosanoic acid	11.1±0.5	-	5.6±0.3
Docosanoic acid, methyl ester	6.3±1.3	-	3.2±0.6
Tetracosanoic acid	5.2±2.5	-	2.6±1.8
Tetracosanoic acid, methyl ester	11.6±5.0	2.3±4.0	7.0±0.7
Hexacosanoic acid	3.3±1.2	-	1.7±0.9
***Substituted alkanoic acids***	***21*.*0±15*.*6***	-	***10*.*5±7*.*7***
9.12-Octadecadienoic acid	1.0±0.5	-	0.5±0.3
9.12-Octadecadienoic acid, methyl ester	16.2±13.6	-	8.1±6.6
*cis*-9-Octadecenoic acid	2.6±1.1	-	1.3±0.6
16-Methyl-heptadecanoic acid. methyl ester	1.2±0.4	-	0.6±0.2
***Saturated ω-hydroxyacids***	***269*.*4±90*.*7***	***107*.*6±89*.*5***	***187*.*7±95*.*5***
16-Hydroxyhexadecanoic acid	9.7±4.3	2.1±3.6	5.9±4.0
16-Hydroxyhexadecanoic acid, methyl ester	9.4±3.8	7.2±2.2	8.3±3.0
18-Hydroxyoctadecanoic acid, methyl ester	50.5±11.3	46.2±37.9	48.4±24.6
20-Hydroxyeicosanoic acid	10.0±3.9	-	5.0±2.0
20-Hydroxyeicosanoic acid, methyl ester	10.9±0.3	-	5.4±0.2
22-Hydroxydocosanoic acid	45.4±4.2	39.8±24.3	42.6±14.3
22-Hydroxydocosanoic acid, methyl ester	29.8±26.8	4.5±7.9	17.2±17.4
24-Hydroxytetracosanoic acid	63.3±15.4	7.8±13.6	35.6±14.5
24-Hydroxytetracosanoic acid, methyl ester	16.3±16.4	-	8.2±8.2
26-Hydroxyhexacosanoic acid	7.7±4.2	-	3.8±2.1
26-Hydroxyhexacosanoic acid, methyl ester	14.6±10.1	-	7.3±5.2
***Substituted ω-hydroxyacids***	***153*.*4±13*.*3***	***398*.*5±76*.*4***	***276*.*1±44*.*8***
16-Methoxyhexadecanoic acid	7.0±1.6	1.6±0.7	4.3±1.2
18-Hydroxy-9-octadecanoic acid, methyl ester	0.0±0.0	88.7±49.6	44.4±24.8
18-Methoxyoctadecanoic acid	14.3±0.5	-	7.2±0.2
18-Hydroxy-9,10-epoxyoctadecanoic acid, methyl ester	52.7±2.0	-	26.4±1.0
18-Hydroxy-9,10-dihydroxyoctadecanoic acid, methyl ester	79.4±9.2	308.2±26.1	193.8±17.6
***Saturated ɑ*,*ω-alkanoic diacids***	***38*.*0±27*.*3***	***2*.*8±1*.*6***	***20*.*5±14*.*9***
Octanedioic acid	4.1±2.7	2.8±1.6	3.4±2.2
Heptanedioic acid	1.3±0.7	-	0.7±0.4
Heptanedioic acid, methyl ester	0.9±0.4	-	0.4±0.2
Nonanedioic acid	5.6±1.1	-	2.8±0.6
Hexadecanedioic acid	3.6±0.6	-	1.8±0.3
Hexadecanedioic acid, dimethyl ester	2.4±0.8	-	1.2±0.4
Octadecanedioic acid, methyl ester	14.3±19.0	-	7.2±9.5
Octadecanedioic acid, dimethyl ester	5.8±2.6	-	2.9±1.3
***Substituted ɑ*,*ω-alkanoic diacids***	***132*.*8±25*.*3***	***272*.*9±83*.*2***	***202*.*9±41*.*0***
9,10-Dihydroxyoctadecanedioic acid, dimethyl ester	42.0±4.4	92.2±13.8	67.1±6.7
8,9,18-Trihydroxyoctadecanedioic acid, methyl ester	90.8±20.9	60.7±34.0	75.8±9.3
2-Hydroxydecanedioic acid	0.0±0.0	120.0±35.4	60.0±25.0
***Sterols***	***3*.*6±1*.*0***	-	***1*.*8±0*.*7***
β-Sitosterol	3.6±1.0	-	1.8±0.7
***Triterpenes/Triterpenoids***	***10*.*4±0*.*7***	-	***5*.*2±2*.*3***
Betulin	0.8±0.1	-	0.4±0.1
Betulinic acid	0.8±0.3	-	0.4±0.2
Dehydroabietic acid	8.8±0.3	-	4.4±2.0
***Identified***	***858*.*7±255*.*0***	***919*.*5±309*.*8***	***887*.*7±273*.*7***
Non Identified	141.3	80.5	112.3
Total	1000.0	1000.0	1000.0

The main constituents found in suberin from *Q*. *faginea* bark are fatty acids (70.7–88.3% of all compounds): substituted ω-hydroxyacids (15.3% in site 1 and 40.2% in site 2), saturated ω-hydroxyacids (26.8% in site 1 and 10.8% in site 2), and substituted ɑ,ω-alkanoic diacids (13.3%in site 1 and 27.3%in site 2). The differences between the barks from both sites are evident: in site 1 the ω-hydroxyacids correspond to 60.5% of total fatty acids and in site 2 substituted ω-hydroxyacids and ɑ,ω-diacids constitute 80.1% of total fatty acids.

Factors such as the local geoclimate, seasonal changes, external conditions such as light, temperature and humidity may affect the composition of secondary metabolites [59] and contribute to the differences on the chemical composition of the barks from the two sites.

In the barks of site 1, suberin is mainly constituted by saturated ω-hydroxyacids including namely 24-hydroxytetracosanoic acid and its methyl ester (10.3%), 22-hydroxydocosanoic acid (6.2%) and the methyl ester of 18-hydroxyoctadecanoic acid (5.0%). saturated and substituted alkanoic acids represented 6.5% and 2.1% of identified compounds (namely docosanoic acid and the methyl esters of hexadecanoic, tetracosanoic and 9,12-octadecanedioic acids). Substituted ω-hydroxyacids and substituted ɑ,ω-diacids were also found in high amounts (15,3% and 13.3%, respectively) where the major compound identified was the methyl esters of 18-hydroxy-9,10-dihydroxyoctadecanoic (7.9%) and 18-hydroxy-9,10-epoxyoctadecanoic acid (5.3%). Alkanols represented 6.8% of all compounds, Saturated ɑ,ω-diacids corresponded to 3.8% (where the methyl ester of octadecanedioic acid exist in 1.4%), Aromatics represent 9.8% (mainly ferulic acid and its methyl ester). Sterols and triterpenes were present in minor amounts.

In the barks of site 2, suberin is richer in substituted ω-hydroxyacids (39.8%) and substituted ɑ,ω-diacids (27.3%), mainly the methyl esters of 18-hydroxy-9,10-dihydroxyoctadecanoic acid (30.8%), 18-hydroxy-9-octadecanoic acid (8.9%), 2-hydroxydecanedioic acid (12.0%) and the methyl esters of 9,10-dihydroxyoctadecanedioic (9.2%) and 8,9,18-trihydroxyoctadecanedioic acids (6.1%). Saturated ω-hydroxyacids were also found in considerable amounts (10.8%), and the methyl ester of 18-hydroxyoctadecanoic acid and 22-hydroxydocosanoic acid are the major identified compounds. Alkanols represented 6.6% of all compounds (namely 1-octadecanol with 2.8%), saturated alkanoic acids 3.2% (mainly hexadecanoic and octadecanoic acids). The methyl ester of ferulic acid was the only aromatic found representing 4.0% of the compounds. Chain lengths ranged from C14 to C26, but C18 was the most relevant, representing 52.2% and 73.1% of the total fatty acids identified in site 1 and 2, respectively.

This is the first report of suberin composition from *Q*. *faginea* bark showing that it is characterized by the major presence of ω-hydroxyacids (46.3% of total compounds) and of ɑ,ω-alkanoic diacids (22.3%). This composition is quite different from that of cork from *Q*. *suber* in which the most important monomers are substituted ɑ,ω- diacids with mid-chain epoxy or diol substitutions [56]. Differences also occur with the suberin of *Q*. *cerris* cork where the ω-hydroxyacids represent 90% of the long chain monomers [37]. A more similar compositional pattern is found with the suberins of *Pseudotsuga menziesii* cork [22] or *Plathymenia coriacea* [60].

It is worth noticing that the contents in saturated fatty acids such as ω-hydroxyacids and alkanoic acids amounts are over twofold in site 1 (younger trees) than in site 2 (older trees), while ɑ,ω-alkanoic diacids and ω-hydroxyacids are the most representative in site 2.

Triterpenes and sterols are absent in site 2. Although the number of samples used in these studies is small and general conclusions have to be made cautiously, the results suggest a possible relation between tree age and suberin chemical composition: in older trees suberin is constituted by higher content of mid-chain substituted fatty acids, leading to a spatially less-compact macromolecular structure [61].

## Conclusions

The barks from *Quercus faginea* mature trees from two sites were chemically characterized for the first time, showing a high content of extractives, constituted mainly by polar compounds extractable with ethanol and water that include high contents of phenolics and polyphenolics, including of flavonoids. The ethanol-water extracts showed a very high antioxidant capacity, well above most reports on other materials.

The bark of *Q*. *faginea* contains a high amount of inorganic material and of lignin. In accordance with its structural composition the bark has a small amount of suberin. Suberin composition is dominated by ω–hydroxyacids (saturated and substituted) and to a lesser extent extent by ɑ,ω-diacids.

In an integrated valorization strategy, *Quercus faginea* barks are interesting sources of polar compounds including phenols and polyphenols with possible interesting bioactivities. The lipophilic extracts contained sterols and triterpenes that are also valuable bioactive compounds or chemical intermediates for the synthesis of new valuable compounds with specific properties.
